# Transcriptomic analysis of the salivary gland of medicinal leech *Hirudo nipponia*

**DOI:** 10.1371/journal.pone.0205875

**Published:** 2018-10-19

**Authors:** Zenghui Lu, Ping Shi, Huajian You, Yanqi Liu, Shijiang Chen

**Affiliations:** 1 Chongqing Academy of Chinese Materia Medica, Chongqing, China; 2 Chongqing Sub-center of National Resource Center for Chinese Materia Medica, China Academy of Chinese Medical Science, Chongqing, China; 3 Chongqing Key Laboratory of Chinese Medicine Resources, Chongqing, China; Institute of Medical Research and Medicinal Plant Studies, CAMEROON

## Abstract

*Hirudo nipponia* (known as Shui Zhi in Chinese) is a well-known Chinese medicine with numerous active ingredients in its body, especially in its saliva. This native Chinese blood-sucking leech has been used for therapeutic purposes since before 100 AD. Modern Chinese physicians use it for a wide range of diseases. Genomic data and molecular information about the pharmacologically active substances produced by this medicinal leech are presently unavailable despite this organism’s medicinal importance. In this study, we performed transcriptome profiling of the salivary glands of medicinal leech *H*. *nipponia* using the Illumina platform. In total, 84,657,362 clean reads were assembled into 50,535 unigenes. The obtained unigenes were compared to public databases. Furthermore, a unigene sequence similarity search and comparisons with the whole transcriptome of medical leech were performed to identify potential proteins. Finally, more than 21 genes were predicted to be involved in anticoagulatory, antithrombotic, antibacterial, anti-inflammatory and antitumor processes, which might play important roles in the treatment of various diseases. This study is the first analysis of a sialotranscriptome in *H*. *nipponia*. The transcriptome profile will shed light on its genetic background and provide a useful tool to deepen our understanding of the medical value of *H*. *nipponia*.

## Introduction

*Hirudo nipponia* Whitman (Shui Zhi in Chinese) is a traditional Chinese medicine with significant medicinal values. It has been widely used to treat cardiovascular and cerebrovascular diseases, hyperlipidemia, thrombosis, inflammation, and tumor diseases in China and was first recorded in the classic book on Chinese Materia Medica, *Shen-Nong-Ben-Cao-Jing* (ca. 100 AD) [1–3]. Now, this leech is mainly used in cerebral thrombosis and cerebral apoplexy in clinical treatment and is listed in the Pharmacopoeia of the People’s Republic of China [4,5]. To date, over 34 active ingredients, including peptides, phosphatidylcholines, pteridines, and other components, have been isolated and/or structurally identified in *H*. *nipponia* [1,6,7,8]. More than 300 Shui Zhi-containing prescriptions are produced by many pharmaceutical manufacturers, including the Maixuekang capsule, Tongxinluo capsule and Da Huang Zhe Chong pill [9–11].

Although this medicinal leech has been used as treatment for various ailments in China, Korea and Japan for a long time, neither its pharmacologically active compounds nor its molecular information has been thoroughly investigated. Next generation sequencing platforms based on the RNA-Seq technique are a powerful and efficient tool to detect novel transcripts.

Therefore, we utilized Illumina de novo sequencing technology to detect the transcriptional profiles of *H*. *nipponia*. The sialotranscriptome described here provides a significant amount of gene resources for a comprehensive understanding of the pharmacologically active compounds produced by this leech. This study also provides useful information on leading compounds with pharmaceutical potential in *H*. *nipponia*.

## Materials and methods

### Animals and RNA extraction

All leeches were obtained from an adult *Hirudo nipponia* colony grown in a medical leech breeding base at the Chongqing Academy of Chinese Materia Medica (Nan’an, China). The 50 leeches were maintained in glass container filled with 15 L dechlorinated tap water at 25°C and 12 h/12 h day/night cycles prior to dissection. Every 5 days, half of the water was removed and replaced with fresh water. Prior to RNA extraction, leeches were washed in 0.5% bleach for 1 min and subsequently rinsed in deionized water for 30 seconds to minimize contamination with bacteria. Salivary tissue masses lying posterior to the 3 muscular jaws from twenty leeches were removed aseptically by sterilized dissecting tool and subsequently rinsed in 0.5% bleach for 1 min followed by rinsing in deionized water for 1 min [12–14].Total RNA was then extracted from the abovementioned salivary tissues using a RNAprep Pure Tissue Kit (Tiangen, China). RNA quality was monitored on 1% agarose gels, and the concentration was determined using a Qubit® RNA Assay Kit in Qubit® 2.0 Flurometer (Life Technologies, CA, USA).

### Library preparation for transcriptome sequencing

Construction of the cDNA libraries and RNA-Seq were performed by Novogene Bioinformatics Technology Co., Ltd. (Beijing, China). First, mRNA was purified from 1.5 μg of total RNA from salivary gland tissue using poly-T oligo-attached magnetic beads. Fragmentation was carried out using divalent cations under elevated temperatures in NEB Next First Strand Synthesis Reaction Buffer (5×). First strand cDNA was synthesized using a random hexamer primer and M-MuLV Reverse Transcriptase (RNase H-). Subsequently, second strand cDNA synthesis was performed using DNA Polymerase I and RNase H. Remaining overhangs were converted into blunt ends via exonuclease/polymerase activities. After the adenylation of 3’ ends of DNA fragments, NEBNext Adaptors with a hairpin loop structure were ligated to prepare for hybridization. The library fragments were purified with the AMPure XP system (Beckman Coulter, Beverly, USA) to select cDNA fragments that were preferentially 150~200 bp in length. Then, 3 μL of USER Enzyme (NEB, USA) was used with size-selected, adaptor-ligated cDNA at 37°C for 15 min followed by 5 min at 95°C before PCR. Then, PCR was performed with Phusion High-Fidelity DNA polymerase, Universal PCR primers and Index (X) Primer. Finally, PCR products were purified (AMPure XP system), and library quality was assessed on an Agilent Bioanalyzer 2100 system.

### De novo transcriptome assembly and functional annotation

The clean reads were obtained by removing reads with adapter sequences, reads containing ploy-N (N>0.1%), and low-quality reads from the raw data. After filtering, the high-quality clean data were de novo assembled by a Trinity RNA-Seq Assembler [15]. Unigenes were matched to publicly accessible databases using Blastx (E-value ≤ 10^−5^), including NCBI redundant protein sequences (Nr), NCBI non-redundant nucleotide sequences (Nt), protein family (Pfam), EuKaryotic Orthologous Groups (KOG), Swiss-Prot Protein Sequence Database (Swiss-Prot), Kyoto Encyclopedia of Genes and Genomes (KEGG), and Gene Ontology (GO) databases.

### Multiple sequences alignments and analysis

All unigenes were converted to their corresponding predicted amino acid sequences with Virtual Ribosome [16]. These putative polypeptide sequences then were used to retrieve orthologous sequences from GenBank nr with blastp and from GenBank EST with tblastn. In addition to global annotations predicted against GenBank nr databases, blastx comparisons were made against a locally compiled sequence database of the following accessions: Q07558 hirudin from *H*. *medicinalis*, P81492 hirudin from *Hirudinaria manillensis*, P15358 antistasin from *Haementeria officinalis*, AAA65645 ghilanten from *H*. *ghilianii*, P80302 hirustasin from *H*. *medicinalis*, AAD09442 guamerin from *H*.*nipponia*, 2K13 saratin from *H*. *officinalis*, AAA96144 destabilase from *H*. *medicinalis*, AAF73890 bdellin-kl from *H*. *nipponia*, P82107 bdellin A from *H*. *medicinalis*, P09865 bdellin B from *H*. *medicinalis*, and etc. Comparative amino acid sequence alignment was accomplished with CLUSTAL W and BoxShade server at heep://www.ch.embnet.org/software/BOX_form.html. Signal peptide prediction was accomplished with SignalP 4.1 server at http://www.cbs.dtu.dk/services/SignalP/.

## Results and discussion

### Transcriptome sequencing and assembly

**Fig 1:**
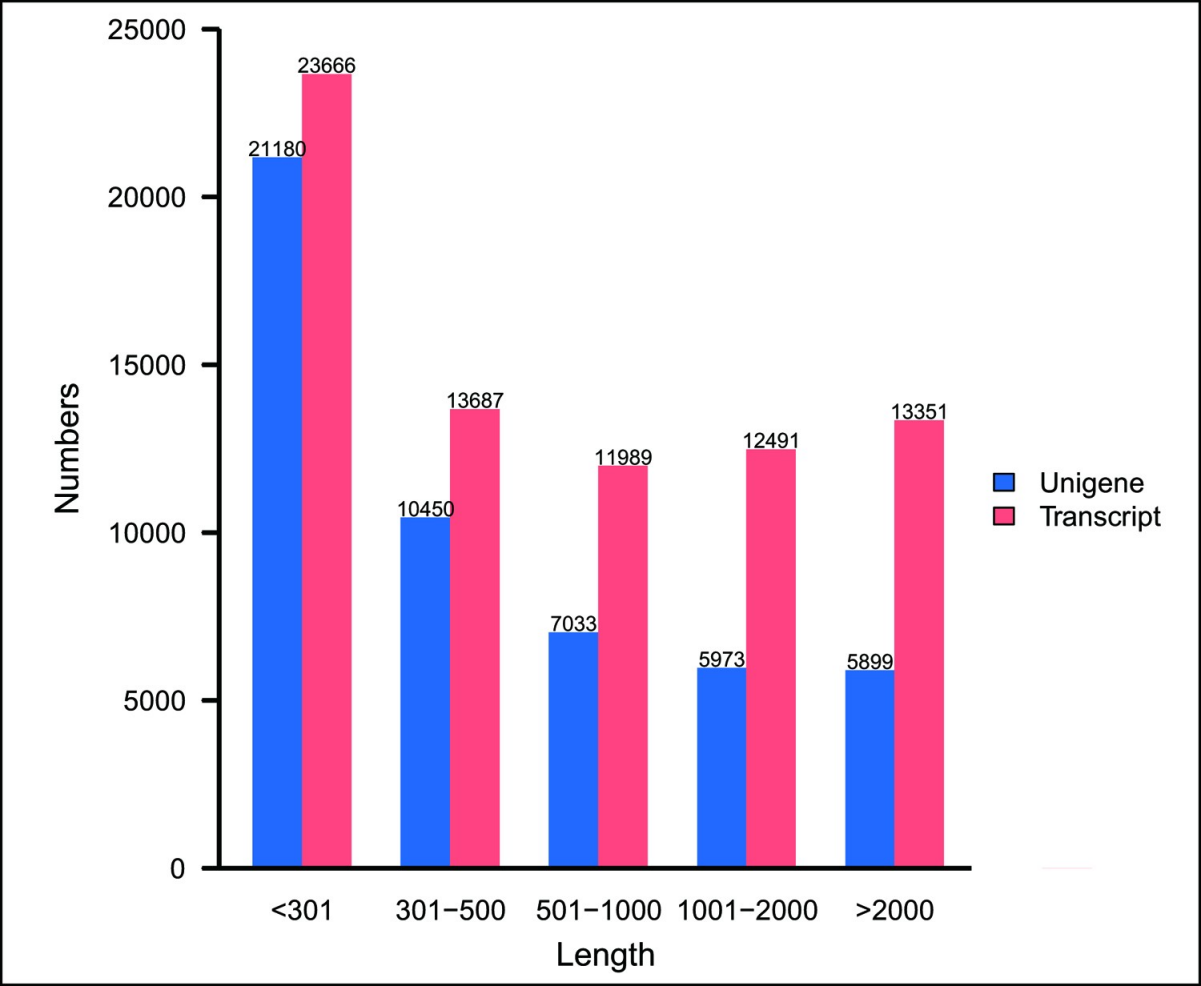
Length distribution of unigenes and transcripts in base pairs.

Illumina sequencing data from the salivary glands of *H*. *nipponia* were deposited to NCBI SRA database under accession number SRP126617. A total of 89,867,368 Illumina paired-end raw reads were identified (Table 1). In total, 84,657,362 clean reads were obtained by filtering out adaptor sequences, ambiguous nucleotides, and low-quality sequences. The assembly of clean reads using Trinity software resulted in 50,535 unigenes that ranged from 201 bp to 28,648 bp with a N50 length of 1,878 bp (Table 1). The length distribution of all unigenes and transcripts is shown in Fig 1.

**Table 1:** Summary of transcriptome data analysis of *Hirudo nipponia*.

Description	Number
Total number of raw reads	89,867,368
Total number of clean reads	84,657,362
Total length of clean reads (Gb)	12.7
GC content (%)	40.21
Q20 percentage (%)	95.66
Q30 percentage (%)	90.60
Total nucleotides of unigenes	42,990,487
Total number of unigenes	50,535
Min length of assembly (bp)	201
Max length of assembly (bp)	28,648
Average length of assembly (bp)	851
N50 length of assembly (bp)	1,878
N90 length of assembly (bp)	276

### Gene annotation of salivary gland

The 50,535 unigene sequences from the salivary gland of *H*. *nipponia* were functionally annotated by searching diverse public databases, including Nr, Nt, Pfam, KOG, Swiss-Prot, KEGG, and GO. Overall, a total of 23,490 unigenes (46.48%) were successfully annotated with this strategy. The percentage of unique sequences annotated based on Nr, Nt, Pfam, KOG, Swiss-Prot, KEGG and GO were 35.99%, 13.54%, 32.91%, 22.56%, 30.66%, 18.74% and 33.08%, respectively (S1 Table).

**Fig 2:**
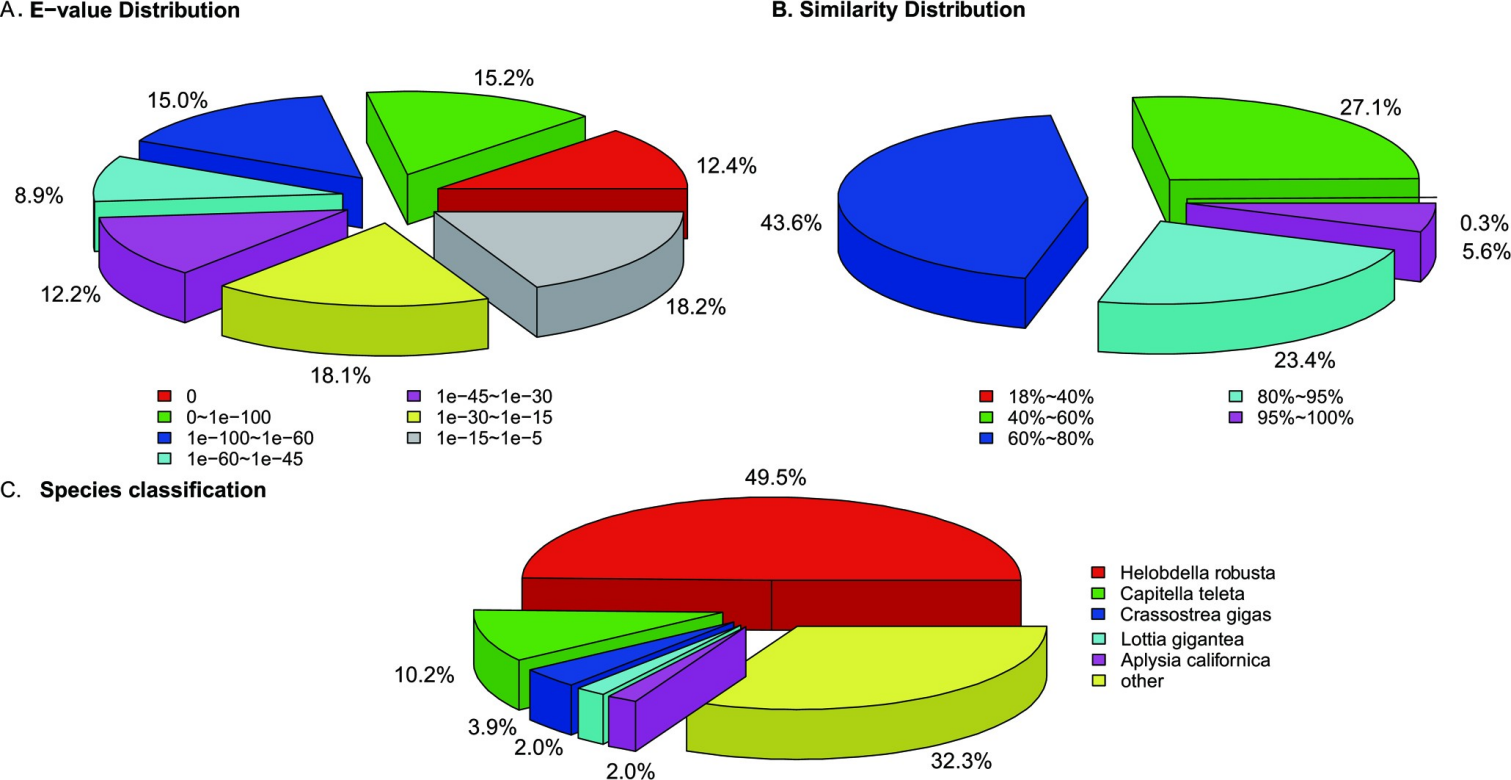
Characteristics of gene annotation according to the Nr database. (A) E-value distribution of Blastx hits for unigenes with a cut-off E-value of 1.0e-5. (B) Similarity distribution of Blastx hits for unigene. (C) Species classification is shown as a percentage of the total homologous sequences with an E-value of at least 1e-5.

The E-value distribution of best hits according to the Nr database revealed that 51.50% of the annotated sequences have strong homology (E-value < 1e-45) and that 48.50% of the homology sequences ranged from 1e-5 to 1e-45 (Fig 2A). The similarity distribution indicated that 72.60% of the annotated sequences had a similarity greater than 60% (Fig 2B). For species classification, the highest percentage of unique sequences matched with leech *Helobdella robusta* (49.5%), followed by the polychaete worm *Capitella teleta* (10.2%) (Fig 2C).

### Functional annotation and pathway assignment

**Fig 3:**
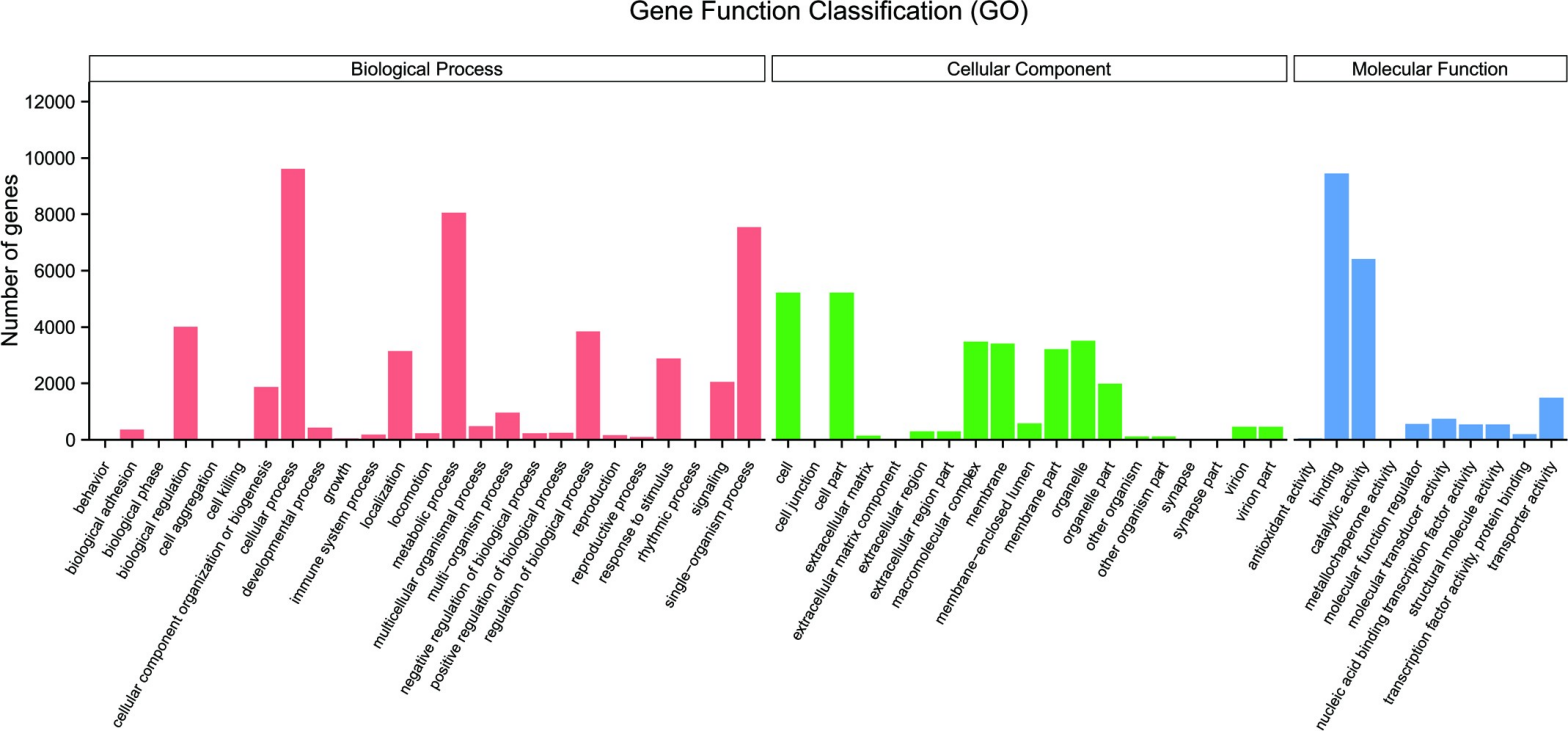
Gene Ontology (GO) categories of genes from *H*. *nipponia* salivary glands. GO functional annotations are summarized in three main categories: biological process, cellular component and molecular function. Each category represents a GO term assigned by Blast2GO analysis.

Based on GO, an international standardized gene functional classification system, 16,718 non-redundant unigenes were assigned to 54 level 2 GO terms, which were classified into three main functional categories, i.e., biological process, cellular component and molecular function (Fig 3).

In the category of biological process function, the terms cellular process (9,617, 20.67%) and metabolic process (8,064, 17.33%) were the dominant subcategories, followed by single-organism process (7,550, 16.23%). In the category of cellular components, a high percentage of genes were assigned to cell (5,224, 18.28%) and cell part (5,224, 18.28%). For molecular function, the two most abundant categories were binding (9,452, 47.25%) and catalytic activity (6,417, 32.08%). (S2 Table).

**Fig 4:**
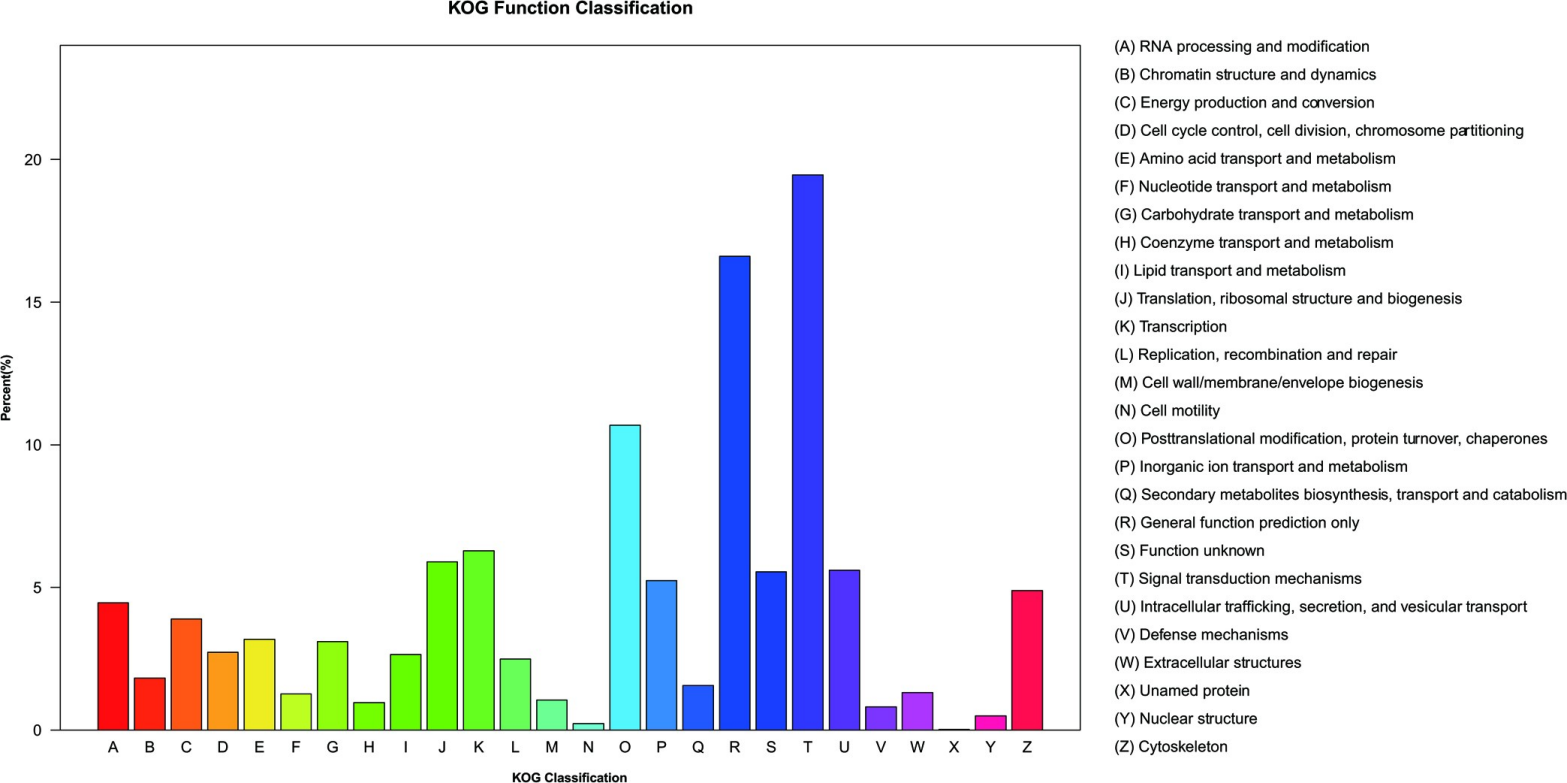
KOG annotation of putative proteins.

Non-redundant unigenes were compared with the KOG database for the analysis of orthologous gene products. A total of 11,404 unigenes with significant homology were assigned to appropriate KOG clusters. These KOG classifications were divided into 26 functional categories. Among them, the cluster of ‘Signal transduction mechanisms’ (19.46%) represented the largest group, followed by ‘General function prediction only’ (16.61%), ‘Posttranslational modification, protein turnover, chaperones’(10.69%) and ‘transcription’ (6.28%) (Fig 4).

The annotated sequences were mapped to the KEGG database to identify the main biological pathways in the salivary gland. In total, 9,471 assembled unigenes in the sialotranscriptome of *H*. *nipponia* were assigned to 230 KEGG pathways. Among the pathways, ribosome, cAMP signaling pathway, MAPK signaling pathway and a few others were highly represented (S1 Fig). The top 20 KEGG pathways are shown in Table 2.

**Table 2:** Top 20 predicted KEGG pathways in the *Hirudo nipponia* sialotranscriptome.

KEGG pathway	Pathway ID	Number of transcripts
Ribosome	ko03010	346
cAMP signaling pathway	ko04024	320
MAPK signaling pathway	ko04010	287
PI3K-Akt signaling pathway	ko04151	286
Calcium signaling pathway	ko04020	256
Endocytosis	ko04144	254
cGMP-PKG signaling pathway	ko04022	254
Oxytocin signaling pathway	ko04921	235
Focal adhesion	ko04510	232
Ras signaling pathway	ko04014	230
Adrenergic signaling in cardiomyocytes	ko04261	228
Oxidative phosphorylation	ko00190	212
Rap1 signaling pathway	ko04015	209
Carbon metabolism	ko01200	208
Purine metabolism	ko00230	207
Regulation of actin cytoskeleton	ko04810	199
Spliceosome	ko03040	195
Neuroactive ligand-receptor interaction	ko04080	193
Protein processing in endoplasmic reticulum	ko04141	192
Thyroid hormone signaling pathway	ko04919	187

### Alignments and genes of interest

The *H*. *nipponia* sialotranscriptome results with high scoring matches revealed a large number of medicinally useful bioactive molecules. Among them, we focused on several proteins potentially related to therapeutic effects, including anticoagulant, thrombolytic, anti-inflammatory, antitumor, anesthetic and vasodilator effects. Anticoagulants included in the locally compiled data set used for the BLASTx comparisons were listed in Table 3.

**Table 3:** Anticoagulants included in the locally compiled data set used for the BLASTx comparisons.

Organism	Bioactive protein	Antagonistic pathway	GenBank accession number/protein	Reference
***Hirudinaria manillensis***	Hirudin-HM1	Thrombin inhibitor	Q07558	[21]
	Hirudin-HM2		P81492	[21]
***Theromyzon tessulatum***	Theromin	Thrombin inhibitor	P82354	[23]
***Hirudo medicinalis***	Hirustasin	Thrombin, trypsin, chymotrypsin, cathepsin G, kallikrein inhibitor	P80302	[24]
***Haementeria officinalis***	Antistasin	Factor Xa inhibitor	AAB29421	[26]
***Haementeria ghilianii***	Ghilanten	Factor Xa inhibitor	AAA65645	[25]
***Hirudo nipponia***	Guamerin	Factor Xa inhibitor (serine protease inhibitor)	AAD09442	[28]
***Hirudo nipponia***	Piguamerin	Trypsin, plasma kallikrein and tissue kallikrein inhibitor	P81499	[29]
***Hirudo nipponia***	Bdellin-KL	Trypsin, plasmin inhibitor	AAF73890	
***Hirudo medicinalis***	Bdellin A		P82107	[32]
***Haementeria officinalis***	Saratin	Thrombocyte aggregation inhibitor	2K13	[38]
***Hirudo medicinalis***	Destabilase Ⅰ	Dissolves stabilized fibrin, stimulates thrombolysis	AAA96144	[39]
***Hirudo medicinalis***	Leech carboxypeptidase inhibitor	Pancreatic and plasma metallocarboxypeptidase inhibitor	P81511	[40]
***Hirudo nipponia***	Hyaluronidase	Antibiotic properties and spreading factor	AHV78514	[46]
***Eisenia andrei***	Lysozyme	Specifically cleaves b-1,4-glycosidic bonds and acts against gram-positive bacteria	ABC68610	
***Theromyzon tessulatum***	Theromyzin	Acts against gram-positive bacteria	Q6T6C1	[49]
***Theromyzon tessulatum***	Theromacin	Acts against gram-positive bacteria	Q6T6C2	[49]
***Hirudo medicinalis***	Neuromacin	Acts against gram-positive bacteria	A8V0B3	[53]
***Salmo salar***	TCTP	Cytokine modulator and virus inhibitor	ACI68930	[50]
***Hirudo medicinalis***	Lumbricin	Acts against fungi, gram-positive and gram-negative bacteria	ABW97520	[53]
***Solen grandis***	C-type lectin	Binds to terminal sugars in microorganisms	AEW43448	[51]

**Fig 5:**
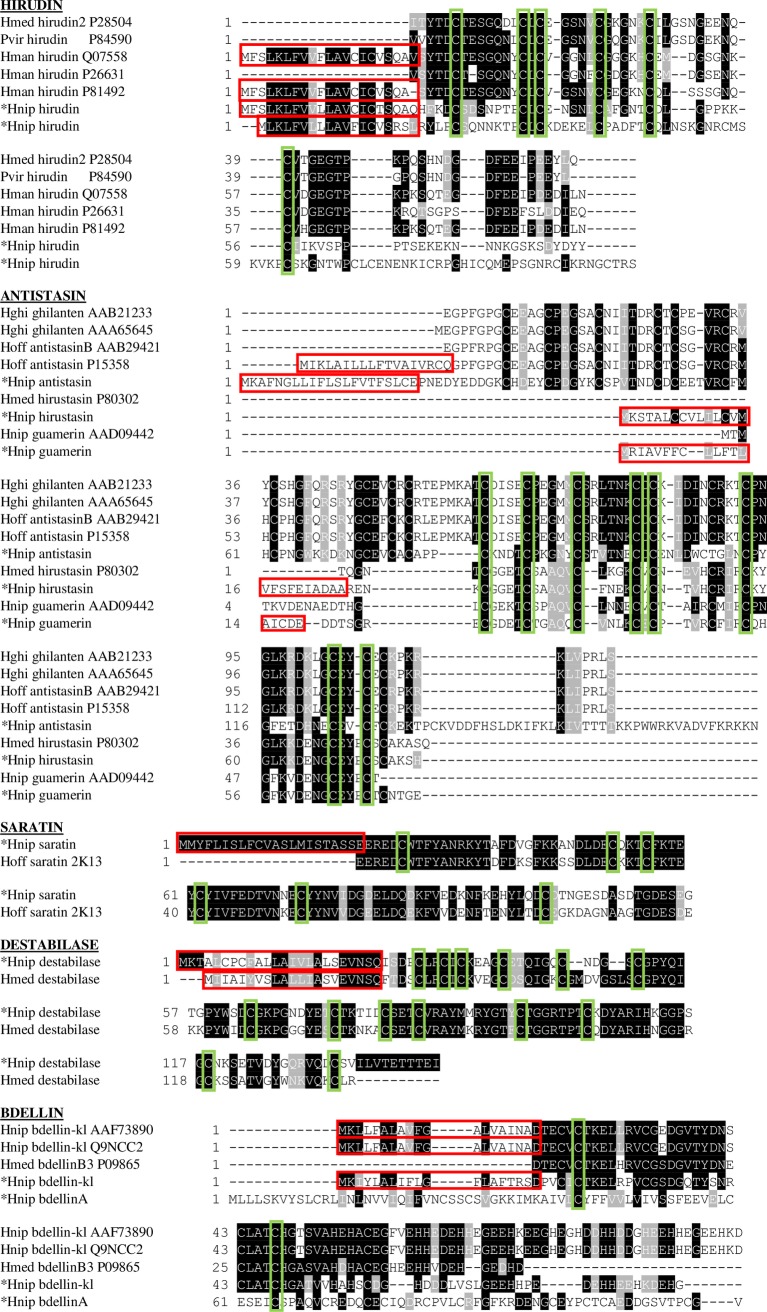
Alignment of inferred amino acid sequences for *Hirudo nipponia* transcripts corresponding to well-characterized leech salivary bioactive peptides. Similar residues are shaded, with the highlighted homology level ranging from dark black (100% identity), black (75–100% identity), grey (50–75% identity), to light grey (33–50% identity). Red boxes correspond to predicted secretory signal peptides. Green boxes outline conserved cysteines. Hnip, *Hirudo nipponia*; Hmed, *Hirudo medicinalis*; Pvir, *Poecilobdella viridis*; Hman, *Hirudinaria manillensis*; Hoff, *Haementeria officinalis*; Hghil, *Haementeria ghilianii*. *The sequences of *H*. *nipponia* described in this study.

**Anticoagulants L**eech salivary glands can produce a diverse pharmacological cocktail of a wide variety of anticoagulants [17]. In the current transcriptome database of *H*. *nipponia*, nine non-redundant unigene sequences involved in anticoagulation and antithrombotic processes showed a high similarity to known leech species. Some of their active ingredients have been isolated and characterized. Hirudin is the most potent natural direct thrombin inhibitor known to date with 65 amino acids, and it can form an irreversible, tight bond to thrombin’s active site [18–20]. Two putative transcripts averaged 52% amino acid identity (E-value = 8.08e-12) and 45% identity (E-value = 8.12e-6) to hirudin from *Hirudinaria manillensis* [21,22]. Several properties of the hirudin “core” motifs associated with hirudin’s binding to the thrombin catalytic pocket are conserved in the putative *H*. *nipponia* sequence, including: CLC, as well as a GSNV region conservatively replaced by chemically similar NSNL in *H*.*nipponia*. All 6 cysteines, presumably involved in 3 disulfide bonds, are evolutionary conserved as well. (Fig 5). Another thrombin inhibitor named theromin was also found in the *H*. *nipponia* sialotranscriptome. The thrombin inhibitor exhibited a high value (K_i_ = 12 fmol/L) for theromin compared with the value for hirudin (K_i_ = 21 fmol/L) [23].

Several serine protease inhibitors, including hirustasin, antistasin, ghilanten, guamerin and piguamerin, were found in the salivary gland. Hirustasin can inhibit trypsin, chymotrypsin, cathepsin G and tissue kallikrein, but it does not inhibit blood coagulation factor Xa activity [24]. In contrast, antistasin and ghilanten are potent inhibitors of factor Xa and are highly homologous to each other [25–27]. Guamerin and piguamerin, which have been purified from *H*. *nipponia* [28,29], were also found in the present sialotranscriptome. Guamerin has a stronger and more specific effect on the inhibition of neutrophilic and pancreatic elastase than piguamerin [28,30]. Furthermore, guamerin can inhibit the release of proinflammatory cytokines (IL-6 & TNF-α) and neutrophil infiltration in cerulein-induced acute pancreatitis [31]. The longest string of conserved amino acid residues across the 9 antistasin (Fig 5) was CxxGLKxDxNGCEY. All 8 cysteines, presumably involved in 4 disulfide bonds, are evolutionary conserved as well (Fig 5). Piguamerin potently inhibits plasma kallikrein, tissue kallikrein and trypsin, but it does not affect the activity of factor Xa, elastase or thrombin [29]. Bdellin-inhibitors, including bdellin A and bdellin-KL, were also blasted successfully according to previously published data [32,33]. They are both non-classical Kazal-type cysteine proteases inhibitors [34,35]. As an inhibitor of trypsin and plasmin, bdellin can exert an anti-inflammatory influence by inhibiting proteases involved in the spread of inflammation [17,36,37]. The longest string of conserved amino acid residues across the 5 bdellins was VCGxDGxTY (Fig 5).

A putative *H*. *nipponia* transcript averaged 75% amino acid identity (E-value = 3.70e-39) with saratin from *Hirudo medicinalis*. The leech protein saratin can prevent thrombocyte aggregation by interfering with the first binding step of thrombocytes to collagen by binding to collagen [38]. The longest strings of conserved residues were EEREDCWTFYANRKYT, and DLDECxKT_55_CFKTEYCYIVFEDTVN (Fig 5). Of these, only Thr_55_ in the alignment corresponds to a residue hypothesized to be involved in this protein’s binding functionality [38]. Destabilase is an isopeptidase that plays a major role in fibrinolytic activity [38] and inhibition of platelet aggregation [41]. Meanwhile, destabilase is also a lysozyme with combined enzymatic and non-enzymatic antibacterial activity [42]. In addition to evolutionary conservation of 14 cysteines, presumably involved in 7 disulfide bonds, there was a 36-residue string of high evolutionary conservation: CSETCVRAYMxRYGTxCTGGRTPTCxDYARIHxGGP (Fig 5). Leech carboxypeptidase inhibitor (LCI) is a potent inhibitor of pancreatic and plasma metallocarboxypeptidases that can be used to treat thrombotic disorders and other cardiovascular diseases [43,44].

**Antibacterial H**yaluronidases, which are glycosidases that predominately degrade hyaluronic acid [45], have a beneficial antimicrobial effect [36]. The hyaluronidases were found in the current *H*. *nipponia* transcriptomic database [46]. It was reported that hyaluronan can facilitate the penetration or diffusion of pharmacologically active substances into body tissues [36,47] and can also enable thrombosis and cancer therapy [46]. Lysozyme is another antimicrobial substance in *H*. *nipponia*, and it had high identity (72%) to a lysozyme in *Eisenia andrei* [48]. In addition, we found two transcripts with similarity to antimicrobial peptides named theromacin and theromyzin. Both transcripts exhibited activity against gram-positive bacteria [49]. A homologue of translationally controlled tumor protein (TCTP) from the salivary gland of *H*. *nipponia* was highly similar to TCTP from *Salmo salara* [50]. TCTP is likely involved in the inhibition of inflammation, immunoregulation and virus prevention by modulating or suppressing cytokines and gene transcription [50,51]. Blastx analysis results showed that a transcript shares 67% sequence identity to lumbricin from *Hirudo medicinalis* and 60% sequence identity to lumbricin-1 from earthworm *Lumbricus rubellus* [52,53]. Lumbricin exhibits strong activity against fungi, gram-positive and gram-negative bacteria, but it does not have hemolytic activity [53]. Another substance, neuromacin, also has antimicrobial activity like lumbricin and theromyzin [53]. C-type lectin is a well-known pattern recognition receptor that can recognize and bind to terminal sugars in microorganisms and participates in immune defense [54,55]. Moreover, both destabilase and bdellin (mentioned above) also have great antibacterial and anti-inflammatory effects.

**Antitumor I**n clinical practice, Shui Zhi and its compound medicines are commonly used to treat a wide range of cancers in China. Previously, published data provided evidence that Shui Zhi could benefit cancer therapy by inhibiting the proliferation of human HepG2 and DNA methylation [1]. Other reports clearly showed that peptides from anticoagulant proteins, such as hirudin and antistasin aforementioned, were metastatic inhibitors against various cancers [56,57]. Since coagulation is related to proliferation and metastasis, blocking the cascade can have an antitumor effect [58,59]. However, such studies are at only a preliminary stage. In addition, there were also over 75 hits with methyltransferases, implying that a potential DNA methylation mechanism probably exists in *H*. *nipponia* [60].

Altogether, the results described above provide direct evidence of the existence of therapeutically active compounds related to anticoagulant, antithrombotic, antibacterial, anti-inflammatory and antitumor effects. Among them, anticoagulatory, antithrombotic and antibacterial substances are the most widely studied, whereas the others are less well-known. Many active ingredients have not been discovered yet due to the absence of molecular evidence in public databases.

## Conclusions

Although *H*. *nipponia* (Shui Zhi) is a potential animal-sourced traditional Chinese medicine with important pharmaceutical value in China, research in this species has been hindered by limited molecular information on its genetic background. In the present study, we sequenced the transcriptome of the primary salivary glands of *H*. *nipponia* using Illumina sequencing technology. The assembled sequence data, comprising 50,535 unique transcripts, provide a value resource for understanding genomic data and the biosynthesis of key bioactive metabolites in *H*. *nipponia*. The present transcriptomic analysis also revealed a series of candidate genes encoding bioactive proteins related to medical treatment. This study has provided a reference for the synthesis of active substances and a valuable resource to further our understanding of the pharmacological mechanisms in *H*. *nipponia*.

## Supporting information

S1 Fig Pathway assignment based on KEGG. (A) Cellular processes categories, (B) Environmental information processing categories, (C) Genetic information processing categories, (D) Cellular processes categories, and (E) Organismal systems categories.
(TIF)

S1 Table Summary statistics for the functional annotation of *Hirudo nipponica* sequences in public databases. (DOCX)

S2 Table The results of GO assignments. (DOCX)
