# Supplementary figures and images for: Transcriptomic analysis of the salivary gland of medicinal leech Hirudo nipponia
Source: PLoS One. 2018 Oct 19;13(10):e0205875. doi: 10.1371/journal.pone.0205875 (PMC6195274; doi:10.1371/journal.pone.0205875)

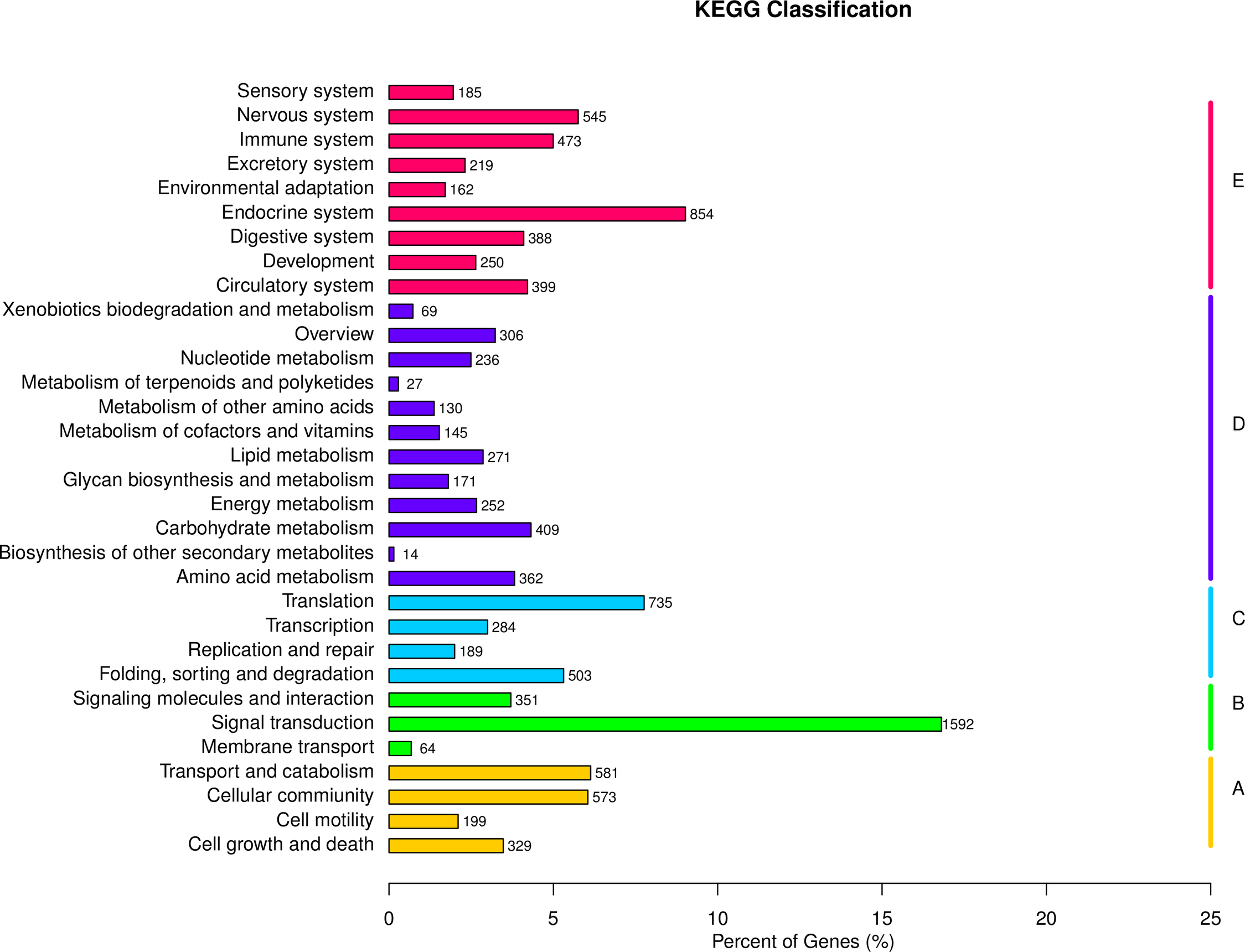

Supplement: S1 Fig — (A) Cellular processes categories, (B) Environmental information processing categories, (C) Genetic information processing categories, (D) Cellular processes categories, and (E) Organismal systems categories. (TIF) [file pone.0205875.s001.tif]
