# Supplementary material for: Transcriptomic analysis of the salivary gland of medicinal leech Hirudo nipponia
Source: PLoS One. 2018 Oct 19;13(10):e0205875. doi: 10.1371/journal.pone.0205875 (PMC6195274; doi:10.1371/journal.pone.0205875)
Supplement: S1 Table — (DOCX) [file pone.0205875.s002.docx]

|  | **Number of Unigenes** | **Percentage (%)** |
| --- | --- | --- |
| **Annotated in NR** | 18191 | 35.99 |
| **Annotated in NT** | 6845 | 13.54 |
| **Annotated in KEGG** | 9471 | 18.74 |
| **Annotated in SwissProt** | 15497 | 30.66 |
| **Annotated in PFAM** | 16633 | 32.91 |
| **Annotated in GO** | 16718 | 33.08 |
| **Annotated in KOG** | 11404 | 22.56 |
| **Annotated in all Databases** | 2701 | 5.34 |
| **Annotated in at least one Database** | 23490 | 46.48 |
| **Total Unigenes** | 50535 | 100 |
