# Supplementary material for: Transcriptomic analysis of the salivary gland of medicinal leech Hirudo nipponia
Source: PLoS One. 2018 Oct 19;13(10):e0205875. doi: 10.1371/journal.pone.0205875 (PMC6195274; doi:10.1371/journal.pone.0205875)
Supplement: S2 Table — (DOCX) [file pone.0205875.s003.docx]

| **GO ID(lev2)** | **GO Term(lev2)** | **GO Term(lev1)** | **Gene Number** |
| --- | --- | --- | --- |
| **GO:0044456** | synapse part | Cellular Component | 9 |
| **GO:0044215** | other organism | Cellular Component | 107 |
| **GO:0032991** | macromolecular complex | Cellular Component | 3487 |
| **GO:0044421** | extracellular region part | Cellular Component | 295 |
| **GO:0044464** | cell part | Cellular Component | 5224 |
| **GO:0044422** | organelle part | Cellular Component | 1981 |
| **GO:0044217** | other organism part | Cellular Component | 107 |
| **GO:0044423** | virion part | Cellular Component | 465 |
| **GO:0044425** | membrane part | Cellular Component | 3213 |
| **GO:0045202** | synapse | Cellular Component | 9 |
| **GO:0044420** | extracellular matrix component | Cellular Component | 3 |
| **GO:0031974** | membrane-enclosed lumen | Cellular Component | 579 |
| **GO:0043226** | organelle | Cellular Component | 3507 |
| **GO:0005623** | cell | Cellular Component | 5224 |
| **GO:0019012** | virion | Cellular Component | 465 |
| **GO:0030054** | cell junction | Cellular Component | 38 |
| **GO:0016020** | membrane | Cellular Component | 3417 |
| **GO:0005576** | extracellular region | Cellular Component | 301 |
| **GO:0031012** | extracellular matrix | Cellular Component | 149 |
| **GO:0098772** | molecular function regulator | Molecular Function | 562 |
| **GO:0005488** | binding Molecular Function | Molecular Function | 9452 |
| **GO:0016530** | metallochaperone activity | Molecular Function | 8 |
| **GO:0003824** | catalytic activity | Molecular Function | 6417 |
| **GO:0016209** | antioxidant activity | Molecular Function | 45 |
| **GO:0005198** | structural molecule activity | Molecular Function | 537 |
| **GO:0000988** | transcription factor activity, protein binding | Molecular Function | 199 |
| **GO:0005215** | transporter activity | Molecular Function | 1494 |
| **GO:0001071** | nucleic acid binding transcription factor activity | Molecular Function | 540 |
| **GO:0060089** | molecular transducer activity | Molecular Function | 751 |
| **GO:0022610** | biological adhesion | Biological Process | 358 |
| **GO:0051179** | localization | Biological Process | 3143 |
| **GO:0048519** | negative regulation of biological process | Biological Process | 230 |
| **GO:0007610** | behavior | Biological Process | 19 |
| **GO:0032501** | multicellular organismal process | Biological Process | 472 |
| **GO:0044848** | biological phase | Biological Process | 16 |
| **GO:0000003** | reproduction | Biological Process | 168 |
| **GO:0009987** | cellular process | Biological Process | 9617 |
| **GO:0071840** | cellular component organization or biogenesis | Biological Process | 1867 |
| **GO:0040007** | growth | Biological Process | 53 |
| **GO:0048518** | positive regulation of biological process | Biological Process | 253 |
| **GO:0002376** | immune system process | Biological Process | 186 |
| **GO:0008152** | metabolic process | Biological Process | 8064 |
| **GO:0050789** | regulation of biological process | Biological Process | 3836 |
| **GO:0044699** | single-organism process | Biological Process | 7550 |
| **GO:0023052** | signaling | Biological Process | 2056 |
| **GO:0050896** | response to stimulus | Biological Process | 2890 |
| **GO:0022414** | reproductive process | Biological Process | 96 |
| **GO:0048511** | rhythmic process | Biological Process | 12 |
| **GO:0001906** | cell killing | Biological Process | 24 |
| **GO:0051704** | multi-organism process | Biological Process | 955 |
| **GO:0040011** | locomotion | Biological Process | 224 |
| **GO:0098743** | cell aggregation | Biological Process | 3 |
| **GO:0032502** | developmental process | Biological Process | 422 |
| **GO:0065007** | biological regulation | Biological Process | 4016 |
